# Detection and Characterization of Clade 1 Reassortant H5N1 Viruses Isolated from Human Cases in Vietnam during 2013

**DOI:** 10.1371/journal.pone.0133867

**Published:** 2015-08-05

**Authors:** Sharmi W. Thor, Hieu Nguyen, Amanda Balish, Anh Nguyen Hoang, Kortney M. Gustin, Pham Thi Nhung, Joyce Jones, Ngoc Nguyen Thu, William Davis, Thao Nguyen Thi Ngoc, Yunho Jang, Katrina Sleeman, Julie Villanueva, James Kile, Larisa V. Gubareva, Stephen Lindstrom, Terrence M. Tumpey, C. Todd Davis, Nguyen Thanh Long

**Affiliations:** 1 Influenza Division, Centers for Disease Control and Prevention, Atlanta, Georgia, United States of America; 2 Institute Pasteur-Ho Chi Minh City, National Influenza Center-2, Ho Chi Minh City, Vietnam; 3 Influenza Program, Centers for Disease Control and Prevention- Vietnam, Hanoi, Vietnam; Centers for Disease Control, TAIWAN

## Abstract

Highly pathogenic avian influenza (HPAI) H5N1 is endemic in Vietnamese poultry and has caused sporadic human infection in Vietnam since 2003. Human infections with HPAI H5N1 are of concern due to a high mortality rate and the potential for the emergence of pandemic viruses with sustained human-to-human transmission. Viruses isolated from humans in southern Vietnam have been classified as clade 1 with a single genome constellation (VN3) since their earliest detection in 2003. This is consistent with detection of this clade/genotype in poultry viruses endemic to the Mekong River Delta and surrounding regions. Comparison of H5N1 viruses detected in humans from southern Vietnamese provinces during 2012 and 2013 revealed the emergence of a 2013 reassortant virus with clade 1.1.2 hemagglutinin (HA) and neuraminidase (NA) surface protein genes but internal genes derived from clade 2.3.2.1a viruses (A/Hubei/1/2010-like; VN12). Closer analysis revealed mutations in multiple genes of this novel genotype (referred to as VN49) previously associated with increased virulence in animal models and other markers of adaptation to mammalian hosts. Despite the changes identified between the 2012 and 2013 genotypes analyzed, their virulence in a ferret model was similar. Antigenically, the 2013 viruses were less cross-reactive with ferret antiserum produced to the clade 1 progenitor virus, A/Vietnam/1203/2004, but reacted with antiserum produced against a new clade 1.1.2 WHO candidate vaccine virus (A/Cambodia/W0526301/2012) with comparable hemagglutination inhibition titers as the homologous antigen. Together, these results indicate changes to both surface and internal protein genes of H5N1 viruses circulating in southern Vietnam compared to 2012 and earlier viruses.

## Introduction

The natural hosts for the majority of influenza A viruses are wild, aquatic birds of the orders Anseriformes (ducks, geese, and swans) and Charadriiformes (gulls, terns, and plovers). However, influenza A viruses can infect a wide range of animals including humans, pigs, poultry, horses, dogs, and marine mammals. Influenza A is a member of the viral family *Orthomyxoviridae* and contains eight segmented, negative-sense RNAs encoding 11 proteins; polymerase basic protein 2 (PB2), polymerase basic protein 1 (PB1), polymerase basic protein 1-F2 (PB1-F2), polymerase acid (PA), hemagglutinin (HA), nucleoprotein (NP), neuraminidase (NA), matrix protein 1 (M1), matrix protein 2 (M2), nonstructural protein 1 (NS1) and nonstructural protein 2 (NS2). Virus classification is determined by the two glycoproteins on the surface of the virus, the HA and NA, of which 18 HA and 11 NA subtypes have been identified [1–3]. Aquatic birds are migratory in nature resulting in the potential for wide geographic spread and distribution of circulating subtypes, increasing the potential for contact with domesticated animals, including poultry, swine and humans.

Highly pathogenic avian influenza (HPAI) H5N1 virus emerged as a human pathogen in 1997 with the infection of a three year old boy in Hong Kong, Special Administrative Region (SAR) [4]. This event raised worldwide concern about H5N1 as a potential zoonotic pandemic threat as all of the eight gene segments of this virus were found to be of avian origin evincing direct avian to human transmission [4,5]. Although human cases of H5N1 infection are sporadic events that occur where the virus is circulating endemically in poultry, the virus remains a public health concern due to its high mortality rate (59%) and the possibility of acquiring sustained human-to-human transmission [6,7].

H5N1 is considered endemic in poultry in Bangladesh, China, Egypt, India, Indonesia, and Vietnam [8]. It is at the interface with domestic poultry where many mammalian species, including humans, are at highest risk for contracting infection. A study of human H5N1 infections reported from 2006 to 2010 found that 96% of all cases were associated with having contact with infected poultry [9]. Reported in Vietnam since 2003, H5N1 has caused 125 reported human cases to date with a mortality rate of 49.6% [7]. There have been multiple introductions of H5N1 into northern Vietnam and the viruses have spread to southern Vietnam [10]. Several recent studies have identified the genetic lineages of H5N1 viruses that have co-circulated in Vietnam and have classified them by clade according to the nomenclature of the WHO/OIE/FAO H5N1 Evolution Working Group and into more than 40 genotypes according to their complete genome phylogenies [10–13]. Clade 1 H5N1 viruses were first detected in 2003 in northern Vietnam. These viruses spread to southern Vietnam in 2004 where clade 1 remained the predominant H5N1 virus isolated through 2013. Subsequently, clade 1 viruses were also found in Cambodia and Thailand where they also became enzootic causing human infection. Between 2005 and 2007, clade 1 was replaced in the northern provinces of Vietnam by viruses from clades 2.3.2 and 2.3.4. Clades 1 and 2.3.4 were detected at similar levels in northern Vietnam, while 2.3.4 spread into central regions between 2008 and 2010. Clade 2.3.2.1 H5N1 viruses were likely introduced into Vietnam between 2009 and 2010 via poultry trade or wild bird migration as this genetic group has spread widely in many Asian and some European countries (e.g., Bangladesh, Bulgaria, China, India, Indonesia, South Korea, Japan, Mongolia, Romania, and Russia) [14–20].

While the recently described viruses belonging to clade 1.1.2 [21] have predominated in poultry in southern Vietnam, clade 2.3.2.1 (all three subgroups; a, b, and c) predominated in northern and central regions through 2013. Characterization of viruses detected in poultry during routine surveillance of northern Vietnamese provinces has resulted in the identification of viruses exhibiting reassortment between the three clade 2.3.2.1 groups, thus generating 3 recently characterized novel genotypes; VN 46–48 [12]. To date, human H5N1 infections in southern provinces of Vietnam have been characterized as HA clade 1.1.2 and genotype VN3. This report describes the human cases detected in Vietnam in 2012 and 2013 and provides evidence for the emergence of novel reassortant H5N1 viruses in the Mekong Delta region of Vietnam during 2013.

## Results

### Epidemiology of human cases of HPAI H5N1 virus in Vietnam during 2012 and 2013

In 2012, four human cases of H5N1 virus infection in Vietnam were reported to the WHO. Three cases were reported from provinces in southern Vietnam, Dak Lak, Kien Giang, and Soc Trang, with the fourth case from the northern province of Thanh Hoa. The case in Dak Lak (A/Vietnam/CD12-92/2012) was a 31 year old male who was involved in the slaughter and consumption of sick poultry. He was hospitalized shortly after the onset of symptoms and subsequently recovered from his illness. In Kien Giang (A/Vietnam/VP12-3/2012), the case involved an 18 year old male who developed symptoms in early January 2012 following exposure to ducks. The infection proved fatal shortly after admission to a local hospital. The Soc Trang case (A/Vietnam/VP12-07/2012) involved a 26 year old pregnant female who had slaughtered and eaten sick chickens. Her infection was fatal despite admission to a local hospital and treatment with oseltamivir shortly after symptom onset. Samples from the newborn infant tested negative for H5N1. The case in Thanh Hoa province (A/Vietnam/CD12-76/2012) was a 22 year old male who was reportedly involved in the slaughter and consumption of ducks. The patient was treated with antiviral drugs and recovered from his infection. He was hospitalized shortly after symptoms arose and subsequently recovered from his illness. In 2013, the WHO reported 2 human cases of H5N1 in Vietnam. Both of these cases were detected in the southern part of Vietnam in the Mekong River Delta, specifically the provinces of Dong Thap and Long An. The case in Dong Thap (A/Vietnam/VP13-28H/2013) was a 4 year old male who developed a fever and other symptoms shortly after his family slaughtered chickens that were purchased in a village that was later confirmed to have had an H5N1 outbreak in poultry. The patient died shortly after the onset of symptoms. The case in Long An (A/Vietnam/VP39/2013) was a 20 year old female who developed a high fever with coughing and difficulty breathing along with headache and fatigue and later recovered.

### Genomic sequencing of human cases of HPAI H5N1 viruses from Vietnam

Viruses from each of the four reported 2012 human cases of HPAI H5N1 were sent to the Centers for Disease Control and Prevention, Influenza Division by the Pasteur Institute, Ho Chi Minh City for virus isolation and characterization. Virus was recovered for only one (A/Vietnam/VP12-3/2012) of the four samples initially, and the full genome was sequenced and characterized. Only partial sequence data for the HA gene was obtained from the clinical material of the samples from Dak Lak, Soc Trang and Thanh Hoa province (A/Vietnam/CD12-92/2012, A/Vietnam/VP12-07/2012 and A/Vietnam/CD12-76/2012, respectively). The partial sequence from both A/Vietnam/CD12-92/2012 and A/Vietnam/VP12-07/2012 is included in the analysis of the HA gene. An isolate of A/Vietnam/CD12-76/2012 was sent to the CDC and the full genome was subsequently sequenced, characterized and included in this analysis. Clinical materials from both of the cases reported in 2013 (A/Vietnam/VP13-28H/2013 and A/Vietnam/VP39/2013) were sent to the Centers for Diseases Control and Prevention, Influenza Division where the full genome for both viruses was sequenced and analyzed.

### Evolution of H5N1 viruses detected in humans

**Fig 1:**
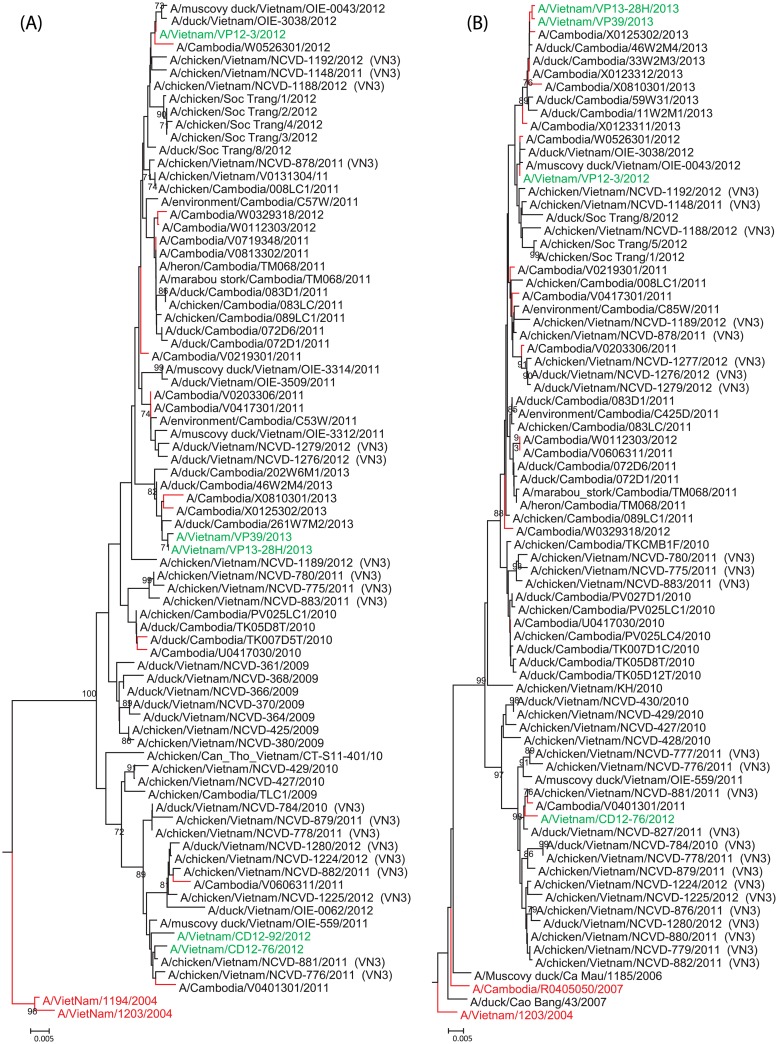
Neighbor-joining phylogenetic analysis of the HA and NA gene. Neighbor-joining phylogenetic tree of the HA (Fig 1a) and NA (Fig 1b) genes of clade 1 highly pathogenic avian influenza A (H5N1) viruses. Red virus strain names denote a WHO candidate reassortant vaccine virus for clade 1. Red branching denotes human cases. The 2012/2013 human cases of H5N1 from Vietnam are denoted by a green strain name. Viruses previously classified with a specific Vietnam genotype are labeled parenthetically with the genotype at the end of the strain name (e.g. VN3, VN12). Bootstraps greater than 70 generated from 1,000 replicates are shown at branch nodes. The scale bar represents nucleotide substitutions per site.

Phylogenetic analysis of the H5 hemagglutinin (HA) was conducted to determine the HA clades of the 2012–2013 viruses isolated from humans in Southern Vietnam. All viruses were found to share a common ancestor with A/goose/Guangdong/1/1996 and belong to either clade 1.1.1 or 1.1.2. The HA genes from the 2012 and 2013 viruses clustered spatially and temporally with related viruses; however A/Vietnam/VP13-28H/2013 and A/Vietnam/VP39/2013 both clustered closely with contemporary viruses on a separate branch (bootstrap value of 71) (Fig 1a). The HA genes from both the 2012 and 2013 viruses were similar to related Vietnamese and Cambodian viruses from either clade 1.1.1 or 1.1.2, as determined by BLAST analysis of the GISAID database (http://platform.gisaid.org/epi3/frontend#20414d) at ≥ 99.3 percent nucleotide identity (% ID) (data not shown). Only one lineage of N1 NA was found in the human viruses isolated during both 2012 and 2013. The clustering of all virus NA genes closely resembled that of the HA phylogeny; with A/Vietnam/VP12-3/2012 and A/Vietnam/CD12-76/2012 clustering with other viruses isolated in Cambodia and Vietnam during 2012 and A/Vietnam/VP13-28H/2013 and A/Vietnam/VP39/2013 grouped with other viruses isolated in 2013 on a separate branch (bootstrap value of 76) (Fig 1b). Using BLAST analysis of the GISAID database (http://platform.gisaid.org/epi3/frontend#20414d), we found all viruses to be highly similar to viruses that cluster with viruses previously identified as from the VN3 genotype at ≥ 99.3% ID (data not shown).

**Fig 2:**
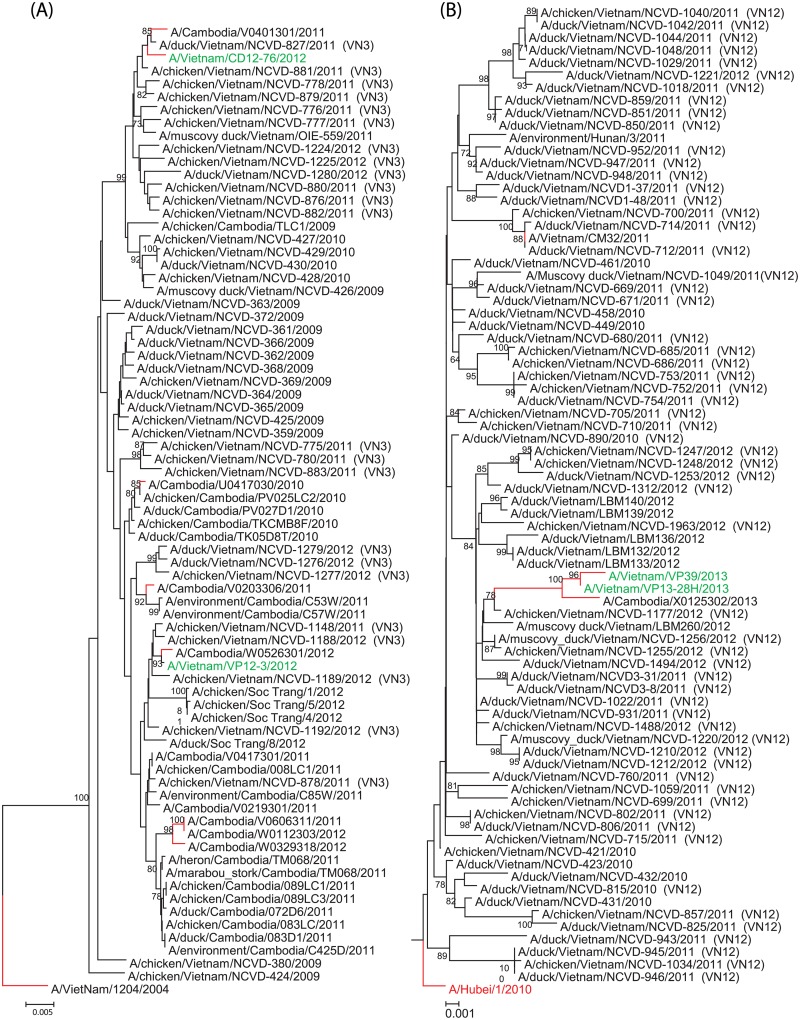
Neighbor-joining phylogenetic analysis of the PB2 gene. Neighbor-joining phylogenetic tree of the PB2 gene of VN3 (clade 1-like, Fig 2a) and VN12 (clade 2.3.2.1a-like, Fig 2b) highly pathogenic avian influenza A (H5N1) virus. Red virus strain names denote a WHO candidate reassortant vaccine virus. Red branching denotes human cases. The 2012/2013 human cases of H5N1 from Vietnam are denoted by a green strain name. Viruses previously classified with a specific Vietnam genotype are labeled parenthetically with the genotype at the end of the strain name (e.g. VN3, VN12). Bootstraps greater than 70 generated from 1,000 replicates are shown at branch nodes. The scale bar represents nucleotide substitutions per site. The phylogenetic groupings for this gene are representative of all internal genes. Phylogenetic analysis of other internal genes can be found in S1–S10 Figs).

Internal gene analysis of A/Vietnam/VP12-3/2012 and A/Vietnam/CD12-76/2012 classified these viruses as genotype VN3 as defined by Wan et al. [10]. Phylogenetic analysis grouped each gene segment with other viruses of this genotype (Fig 2a), and BLAST analysis of the GISAID database (http://platform.gisaid.org/epi3/frontend#20414d) showed each gene segment to have ≥ 99.3% ID with viruses having gene constellations consistent with VN3 [10,12,13](data not shown). The analysis of internal genes from 2013 isolates revealed evidence of a reassortment event between H5N1 viruses of genotype VN3 and VN12. Each of the internal genes from A/Vietnam/VP13-28H/2013 and A/Vietnam/VP39/2013 were closely related to genes found in 2.3.2.1a lineage (Hubei-like) viruses with high nucleotide identity to other viruses of genotype VN12 (≥99.2%). This gene arrangement differed from the isolates obtained in 2012 where all gene segments were related to ancestral clade 1 viruses (Fig 2b).

### Molecular Characterization

Molecular analysis of the amino acid sequence of each virus protein is a critical step in correlating the virus genotype with phenotypic changes. Therefore, each gene of the 2012 and 2013 viruses characterized in this study were analyzed for mutations potentially affecting viral phenotype. A highly pathogenic avian influenza virus multibasic cleavage site motif sequence of PQRERRKKR↓G was present in the HA protein for viruses collected in 2012 as well as 2013 [22]. All viruses have five glycosylation sites at positions 10, 11, 23, 154, and 165. Only A/Vietnam/VP13-28H/2013 and A/Vietnam/VP39/2013, as well as the other viruses isolated in 2013 (S1 Table), have an asparagine (N) at position 155. The 155N mutation has been correlated to increased pseudovirus binding of α-2,6 sialoglycan cellular receptors [23]. Residue number 155 is located in antigenic site B on the globular head of the HA protein. The NA of both the 2012 and 2013 human H5N1 viruses from Vietnam have a deletion of 20 amino acid residues in the stalk region common to other HPAI H5N1 viruses; residues 49 to 68. This deletion has been associated with enhanced virulence in mice [24,25]. Three viruses (A/Vietnam/VP12-3/2012, A/Vietnam/VP13-28H/2013, and A/Vietnam/VP39/2013) had a substitution of NA-V149A (N2 numbering; V129A by H5N1 numbering). When detected in Clade 1 viruses, this substitution was associated with potentially reduced susceptibility to zanamivir [26]; viruses carrying V149A showed slightly elevated zanamivir IC_50_ in the NA inhibition assay (Table 1).

**Table 1:** Susceptibility of highly pathogenic avian influenza A(H5N1) viruses to neuraminidase inhibitors.

		IC_50_ (nmol/L)^b^			
Virus	NA mutation^a^	Oseltamivir	Zanamivir	Peramivir	Laninamivir
A/Vietnam/12-3/2012	149A	0.12	1.49	0.10	0.23
A/Vietnam/CD12-76/2012	149V	0.06	0.32	0.11	0.13
A/Vietnam/VP13-28H/2013	149A	0.12	1.32	0.09	0.16
A/Vietnam/VP39/2013	149A	0.09	0.78	0.08	0.13

^a^ Amino acid residue numbering is based on the H5N1 NA protein.

^b^ IC50: fifty percent inhibitory concentration.

The PB2 protein of A/Vietnam/VP12-3/2012 has a lysine at position 627 (627K) whereas A/Vietnam/CD12-76/2012 has a glutamic acid (E). A lysine at position 627 (627K) is not found in avian isolates of HA clade 1 but sporadically occurs in viruses isolated from humans; the avian isolates predominately have a glutamic acid (E). The mutation E627K is considered a mammalian adaptation [27,28] and has been observed in other human infections of avian influenza A viruses [29,30]. Both 2012 viruses have an aspartic acid at position 701 (701D), which is also thought to be an avian host-specific marker. The isolates from 2013, A/Vietnam/VP13-28H/2013 and A/Vietnam/VP39/2013, had both putative avian host specific markers; 627E and 701D. Analysis of the PB1 protein revealed A/Vietnam/VP12-3/2012 to have not only a full length PB1-F2 (90 amino acids) but also a mutation at position 66 (66S). A serine (S) at position 66 in the PB1-F2 protein has been found by Schmolke et al., to increase virulence and antivirus response in mice [31]. A/Vietnam/CD12-76/2012 does not code for a full length PB1-F2 protein; the protein is truncated at position 25. Both viruses collected in 2013 have a PB1 gene encoding a full length PB1-F2 protein with no mutations identified as altering viral phenotype. The M2 protein for both of the 2012 viruses has S31N substitution in the M2 transmembrane domain, which is a molecular marker of resistance to amantadine and rimantadine [32]. Both of the isolates from 2013 had no known markers of antiviral resistance. The NS protein for both the 2012 and 2013 viruses had a deletion of residues 80–84, a glutamic acid (E) at position 92, and a PDZ ligand motif of ESEV; all of which are characteristics of increased virulence in mice [33–35] (S2 Table).

### Pathotyping in ferrets

We used the ferret model to evaluate the relative virulence of the selected H5N1 viruses, A/Vietnam/VP12-3/2012 and A/Vietnam/VP13-28H/2013. To serve as a comparison, a representative seasonal H3N2 virus, A/Nanchang/933/95 was included. Six ferrets were inoculated intranasally with 10^6^ plaque-forming units (pfu) of each virus and three ferrets were followed for 14 days to monitor clinical symptoms as well as duration of viral shedding. To determine whether the H5N1 viruses were able to replicate in lung and brain tissues, three ferrets were humanely euthanized on day 3 post-inoculation (p.i.) and tissues were collected. Nasal washes were sampled at 1, 3, 5, and 7 days p.i. and titered for the presence of infectious virus. Nasal wash titers peaked for both H5N1 viruses at day 1 p.i. with titers of 10^5.9^ pfu for the 2012 virus and 10^6.7^ pfu for the 2013 viruses (Table 2). In contrast to H3N2 virus-infected ferrets, infectious virus was found in the brains and lungs at relatively high titers in each H5N1-infected ferret, indicating systemic infection. Both 2012 and 2013 H5N1 viruses caused severe disease resulting in death or humane euthanasia in all infected ferrets. This was accompanied by lethargy and substantial mean maximum weight loss of 19 to 22% (Table 2).

**Table 2:** Pathogenicity of clade 1.1 H5N1 viruses in ferrets.

	Clinical signs and virus replication^a^				
Virus	% Weight loss^b^	NW titer^c^	Lethality^d^	Lung titers^e^	Brain titers^e^
A/Vietnam/VP12-3/2012	19.6	5.9 (1)	3/3	6.0 ± 0.5	2.7 ± 1.1
A/Vietnam/VP13-28H/2013	22.6	6.7 (1)	3/3	5.2 ± 1.3	3.3 ± 0.8
A/Nanchang/933/95 (H3N2)	5.1	7.5 (1)	0/3	nd	nd

^a^ H5N1 virus infection was compared to a seasonal H3N2 virus infection. All ferrets were inoculated with 10^6^ PFU of virus.

^b^ The percentage mean maximum weight loss (n = 3) observed during the first 10 days post-inoculation (p.i.)

^c^ Peak mean log_10_ PFU/ml nasal wash titer (day p.i. in parentheses).

^d^ Number of animals euthanized before the end of the 14 day experimental period because of reaching a clinical end point.

^e^ Tissue homogenates from 3 ferrets (day 3 p.i.) were titrated, and the results are expressed as the mean log_10_ PFU/g ± standard deviation.

nd = not detected.

### Antigenic Characterization

The HA inhibition assay was used to assess the antigenic relatedness of the four isolates from 2012 and 2013 human cases in Vietnam. Antiserum against the clade 1 vaccine candidate, A/Vietnam/1203/2004, reacted with the 2012 clade 1.1.1 human isolate with HI titers equivalent to homologous virus titers, but at titers 4 to 8 fold lower than homologous titers for the 2012/2013 clade 1.1.2 human viruses indicating more substantial antigenic drift in viruses belonging to this group of viruses compared to earlier strains. Ferret antiserum raised against clade 1.1, 1.1.1 and 1.1.2 reference viruses (A/duck/Vietnam/NCVD-016/2007, 1.1; A/chicken/Vietnam/NCVD-876/2011, 1.1.1; A/chicken/Vietnam/NCVD-775/2011, 1.1.1; A/Vietnam/VP12-3/2012, 1.1.2) inhibited HA of the 2012 and 2013 with titers that were generally within 2 fold or less to the homologous virus titer (Table 3). Antiserum produced to the recently proposed A/Cambodia/W0526301/2012-like clade 1.1.2 WHO candidate vaccine virus reacted with the clade 1.1.2 viruses with equivalent HI titers but did show a 4-fold drop in titer compared to earlier clade 1.1 and 1.1.1 viruses tested. As expected, ferret antiserum raised against non-clade 1 viruses did not react well with any of the 2012/2013 human Vietnam viruses, exhibiting HI titers that were ≥16-fold lower than the homologous virus titers (Table 3).

**Table 3:** Hemagglutination inhibition reactions of HPAI H5N1 viruses.

		Reference ferret antiserum										
		1	1	1.1	1.1.1	1.1.2	1.1.2	1.1.2	2.1.3	2.2.1	2.3.2.1a	2.3.4
**Reference antigens**	**Clade**	VN/1203	CB/R0405050	DK/VN/16	CK/VN/876	CK/VN/775	VN/VP12-3	CB/W0526301	IND/5	EG/321	CH/1 RG30	ANH/1
A/Vietnam/1203/2004	**1**	**320**	80	160	160	160	320	160	40	<10	160	80
A/Cambodia/R0405050/2007	**1**	40	**160**	20	20	10	40	40	<10	10	20	20
A/duck/Vietnam/NCVD-016/2007	**1.1**	160	40	**80**	40	160	160	80	20	10	40	40
A/chicken/Vietnam/NCVD-876/2011	**1.1.1**	160	40	80	**80**	160	80	80	20	10	40	20
A/chicken/Vietnam/NCVD-775/2011	**1.1.2**	160	20	80	40	**160**	80	80	20	<10	20	20
A/Vietnam/VP12-3/2012	**1.1.2**	80	20	80	20	160	**320**	320	20	<10	10	20
A/Cambodia/W0526301/2012	**1.1.2**	80	20	80	40	80	320	**320**	20	<10	10	20
A/INDONESIA/5/2005	**2.1.3**	40	40	10	10	10	20	40	**640**	40	40	160
A/EGYPT/321-NAMRU3/2007	**2.2.1**	80	40	20	20	<10	10	20	160	**320**	40	80
A/HUBEI/1/2010 IDCDC-RG30	**2.3.2.1a**	40	160	20	20	10	20	40	160	40	**640**	40
A/ANHUI/1/2005	**2.3.4**	20	20	20	80	80	40	40	20	20	10	**640**
**Test antigens**												
A/Vietnam/CD12-76/2012	**1.1.1**	320	40	160	40	160	160	80	40	10	40	20
A/Vietnam/VP13-28H/2013	**1.1.2**	40	20	40	20	80	160	320	20	<10	<10	40
A/Vietnam/VP39/2013	**1.1.2**	80	10	80	40	80	320	640	20	10	10	40

## Discussion

Poultry outbreaks have been recorded in Vietnam since 2003 resulting in a significant number of human cases; 125 cases detected since 2003 (20% of the global total as of Dec 2013). Control measures, such as extensive culling and vaccination of poultry, have been in place for many years to control and reduce the spread in Vietnam. The success of these measures requires systematic outbreak response coupled with active surveillance in affected regions. In 2012, 48 poultry outbreaks along with 4 human cases of HPAI H5N1 were reported to OIE [21] and WHO (WHO, 2013). The majority of the poultry outbreaks occurred in the northern provinces of Vietnam (44 outbreaks/48 total outbreaks; 96%). However, the majority of human cases (3 of 4 cases) were reported from southern provinces. Of the human cases reported from southern Vietnam to date, over half occurred in the Mekong River Delta region. The number of poultry outbreaks of HPAI H5N1 reported to OIE during 2013 dropped to 24 [21] and, unlike the previous year, the majority of outbreaks (23 of 24) were reported from southern provinces. While poultry outbreaks in the Mekong River Delta region only accounted for 25% of the total poultry outbreaks in the country, all of the human cases during 2013 were reported from that area. In poultry, clade 2.3.2.1 (VN12 genotype) has been shown to be the predominately circulating clade in the northern and central regions of Vietnam while clade 1.1 (VN3 genotype) predominates in the southern regions of Vietnam [12]. This observation does not hold true for human cases of H5N1. Although the genotype of a 2011 northern Vietnam case of human H5N1 (A/Vietnam/CM32/2011) [36] is VN12, this is the exception rather than the rule as all other cases to date have been identified as belonging to genotype VN3.

To understand how the molecular signatures associated with these genotypes differed, sequence analysis of each virus protein was carried out. The viral HA protein is a known determinant of host range as it mediates virus binding to specific host cellular receptors; specifically sialic acid receptors [37–39]. The receptor affinity of influenza viruses may vary with the type of host from which the virus is isolated. Influenza viruses of avian origin preferentially bind to α-2,3-linked sialic acid receptors (α-2,3 SA), while mammalian influenza viruses, including human, recognize α-2,6-linked sialic acid receptors (α-2,6 SA). A switch in receptor specificity from avian α-2,3 SA to mammalian α-2,6 SA is one of a number of requirements for an avian virus to become transmissible to mammals. The hallmark of HPAI H5N1 virus is possession of a multibasic cleavage site (MBCS). The MBCS can be cleaved by ubiquitously expressed host proteases facilitating systemic virus replication and up to 100% mortality in poultry [40,41]. Each H5N1 virus isolated from humans during 2012 and 2013 had this motif. Although each of the HA genes can be phylogenetically described as clade 1 (specifically clades 1.1.1 and 1.1.2) there was one mutation consistently different when comparing the 2012 with the 2013 viruses; HA-S155N (H5 numbering; 158N H3 numbering). HA-155N is only present in the 2013 isolates. This amino acid position is located in antigenic site B and is found in a glycosylation motif that is present for all viruses studied herein. Although the motif differs between the 2012 and 2013 isolates, NST and NNT respectively, the mutation is likely to affect the virus by altering the structural shape of the glycosylation site [42,43] not glycosylation itself. Despite the conservation of the S155N change detected in the 2013 viruses, hemagglutination of red blood cells by these viruses was still well inhibited by ferret antibody produced against viruses with serine at this position. Additional studies will be needed to understand how this substitution influences receptor binding and/or antigenicity.

Markers previously associated with reduced susceptibility to antivirals were found in the viruses characterized in this study. Two classes of antiviral drugs are currently available to treat patients with influenza A infection; those that inhibit the enzyme activity of the NA (i.e., oseltamivir and zanamivir) and those that block the M2 ion channel (i.e., amantadine and rimantadine). Of the viruses included in this study, 100% of the viruses isolated in 2013 encoded the mutation V129A (H5N1 numbering), while the mutation was seen in only 50% of the 2012 viruses. The mutation was found in the human cases in southern Vietnam but not the 2012 northern Vietnam case. The effect of V129A mutation on susceptibility to zanamivir is not known, however, it was suggested that this substitution may reduce susceptibility to zanamivir in Clade 1 viruses [26]. In the present study, viruses with this substitution showed only a slight increase in zanamivir IC_50_ which according to the WHO-AVWG criteria [44] is interpreted as *normal inhibition* by zanamivir. It remains unclear whether this mutation occurred spontaneously or as a result of treatment of patients with NA inhibitors. A mutation known to incur resistance to the class of antivirals known as adamantanes, S31N [32], was only found in the amino acid sequence of the M2 protein from the viruses isolated in 2012 (VN3). This mutation has been commonly found in clade 1 H5N1 viruses isolated in Cambodia as well [45]. Adamantanes have been used extensively worldwide in both humans and animals and resistance to this class of antiviral drugs may emerge quickly. Our results suggest that the 2012 H5N1 human viruses (clade 1; VN3) are resistant to adamantanes, while 2013 human viruses (clade 2.3.2.1, VN49) are susceptible due to acquisition of the M gene segment by this new genotype.

Other molecular markers that have been identified as affecting virulence in, as well as adaptation to, mammals were detected in PB2, PB1, and NS1. There are two amino acid positions in PB2 that have been shown to affect the growth of H5N1 influenza viruses in mammalian cells; PB2-627 and PB2-701. Only one of the four viruses in our study had the PB2-627K mutation sometimes found in H5N1 viruses following human infection, A/Vietnam/VP12-3/2012, and 4 of 4 of the viruses had PB2-701D [28,46]. A/Vietnam/VP12-3/2012 was isolated from the throat swab of a patient that would later succumb to the infection. PB2-627K has been identified as an important mutation in facilitating the replication of the virus in the mammalian upper respiratory tract at lower temperatures and as a virulence determinant in mice [27,28,47,48]. PB2-701 has also been implicated in playing a role in virulence and mammalian adaptation [46] with PB2-701N as the molecular marker for H5N1 adaptation to humans and PB2-701D mostly found in avian viruses [49]. A study of human H5N1 infections in northern Vietnam between 2003 and 2008 demonstrated that for H5N1 viruses from clade 1, the combination of mutations at PB2-627 (E or K) and/or PB2-701 (D or N) were not good predictors of either virulence in mice or humans. However, in previous Vietnamese H5N1 viruses isolated from clade 2.3.4 human infections, PB2-627K correlated well with greater virulence in mice despite all examined viruses possessing PB2-701D and was often associated with lethal infection in humans [50]. The viruses studied herein did not indicate that the VN3 (clade 1) or the VN49 (clade 2.3.2.1a) lineage PB2 genes were more or less prone to mutation leading to PB2 substitutions associated with mammalian adaptation.

The over production of inflammatory cytokines and chemokines, systemic viral spreading, and severe multi-organ damage are hallmarks of HPAI-H5N1 infections in both the avian and mammalian host [51,52]. The PB1-F2 protein is present in the majority of avian influenza isolates of all subtypes but the full length protein may have been lost over time in many mammalian isolates [53]. The PB1-F2 protein was first described as a pro-apototic protein that hastened the death of immune cells [54], but has also been shown to play an important role in determining the degree of virulence in mice as an immunomodulator [55]. Each of the 2012/2013 human viruses isolated in southern Vietnam had an intact PB1-F2 protein (90 amino acids) and resulted in a fatal infection. Enhanced pdmH1N1 viral replication in human cells was seen experimentally when PB1-F2 was associated with a mutation of a serine (S) at PB1-F2 position 66 (PB1-F2-66S) [56]. Schmolke et al. consistently observed an increase in both viral replication in murine cells infected with an H5N1 virus expressing the PB1-F2-66S mutation and enhanced virus replication in vivo [31]. In MX1+ mice, only the PB1-F2-66S virus was able to spread to the central nervous system with detectable brain titers at 8 days p.i. [31]. The protein amino acid sequence comparison of the 2012 and 2013 viruses revealed that only A/Vietnam/VP12-3/2012 encoded the PB1-F2-66S mutation. Although experimental evidence has defined some the phenotypic effects of PB1-F2 and the PB1-F2 66S mutation, much of the role of PB1-F2 remains unknown. This study further supports the association of a functional PB1-F2 protein with increasing virulence in mammals.

The NS1 protein for all of the human H5N1 viruses isolated in Vietnam during 2012 and 2013 possessed multiple mutations and/or sequence motifs that have been experimentally shown to enhance virulence. Each protein had a deletion of residues 80–84, a glutamic acid at position 92 (NS1-92E) [33,34], and the presence of four C-terminal residues that form a PDZ domain ligand of the X-S/T-X-V type; ESEV [35]. The deletion of residues 80–84 allow the virus to resist the host antiviral effects of type I IFNs *in vitro* [57] but did not result in increased virulence *in vivo* unless the D92E mutation was present [33]. The NS1-92E mutation can be associated with increased pathogenicity, prolonged viremia, and enhanced clinical signs in both pigs and mice [34,58]. The type I PDZ binding domain found on the C-terminal end of most H5N1 virus NS1 proteins is a motif with the ability to associate with numerous cellular targets. These associations can directly affect the severity of the disease by the modulation of apoptosis and disruption of cellular tight junctions but may act in a strain and host dependent manner [35,59,60]. The NS1 mutations found in the amino acid sequence of all 2012/2013 human H5N1 viruses from Vietnam provide further evidence of the pivotal role NS1 may play in the adaptation of H5N1 viruses to mammalian hosts including humans.

The ability of the virus to not only cause illness in humans but also gain the capacity for sustained human-to-human transmission could result in a pandemic of massive proportion in a population that lacks neutralizing antibodies. Understanding the pathogenicity of avian influenza H5N1 viruses in a mammalian species is of utmost importance if we are to predict the pandemic potential of newly emerging viruses. Ferrets are considered to be the most suitable animal model to study the pathogenesis of avian influenza viruses, including H5N1. In particular, this species exhibits clinical symptoms following influenza virus infection that closely models influenza infection of humans [61]. We evaluated two of the H5N1 viruses isolated from humans in Vietnam; one collected during 2012 (A/Vietnam/VP12-3/2012) and the other collected in 2013 (A/Vietnam/VP13-28H/2013). The HA genes of both of these viruses phylogenetically grouped in the recently described clade 1.1.2 and caused a lethal human infection despite the internal gene differences between the two genotypes. Ferrets infected with the study viruses exhibited classical clinical signs of a HPAI infection: e.g., weight loss, elevated temperature, and extreme lethargy. Each virus replicated to high titers in the respiratory tract, was recovered from multiple organs, including brain tissue, and was lethal. Other clade 1 viruses have reacted similarly in the ferret model [62]. In a comparison of H5N1 viruses isolated in Asia since 1997, Maines et al. showed a pattern of increasing virulence and mortality among the 2004/2005 human H5N1 isolates [62]. A remarkable change in virulence can be caused by a minimum of number amino acid differences [63]. Although the amino acid changes were significant between the 2012 and 2013 human cases from Vietnam, owing to the 2013 viruses having reassorted with a different circulating strain, the differences were insufficient to elicit a remarkable change in virulence and pathogenicity.

This study provides evidence for not only the presence and circulation of HA clade 2.3.2.1a viruses in southern Vietnam but also their ability to successfully reassort with HA clade 1.1.2 viruses and infect humans. As all of the viruses isolated from humans in 2013 were of the reassortant genotype, the maintenance of the VN3 HA and NA with VN12 internal genes may provide the virus with an evolutionary advantage. Phylogenetic analysis of sequence data from public databases and a recent study suggest viruses with this gene constellation have circulated in Cambodia as well (Figs 1 and 2 and S1–S10 Figs) [64]. Although the viruses from 2013 were reassortants, they remain antigenically related to the viruses isolated in 2012 in Vietnam and Cambodia. Continued analysis of human viruses from Vietnam will facilitate the evolutionary monitoring of these viruses and further mutations that may significantly alter the phenotype. There remains limited evidence of human-to-human H5N1 transmission, however there is increasing evidence of H5N1’s ability to cross the species barrier and infect man [65,66]. Neither A/Vietnam/VP12-3/2012 nor A/Vietnam/CD12-76/2012 showed signs of reassortment; both viruses are genotype VN3 similar to all other previous clade 1 viruses. However, both A/Vietnam/VP13-28H/2013 and A/Vietnam/VP39/2013 are progeny derived from a recent reassortment with genotype VN12 with clade 1.1.2 viruses co-circulating in southern Vietnam. Therefore, we suggest this novel genotype be referred to as VN49. Although viruses with the clade 2.3.2.1a HA gene (genotype VN12) continue to show rapid evolution forming distinct subgroups in Bangladesh, Nepal, and Vietnam, the success of the VN49 genotype viruses may lie in an unrecognized internal protein function. The effect of this reassortment on the circulation of this virus will be further evaluated based on longitudinal data from continued surveillance.

HPAI H5N1 virus remains a threat to animal populations, including humans. The continued surveillance and analysis of this virus in poultry and wild birds is especially important in countries, such as Vietnam, where the virus is endemic and the amount of genomic diversity is high. The extent of circulation of VN49 in poultry and/or wild bird populations in Vietnam or elsewhere is currently unknown, however, a recent study has confirmed that this genotype resulted in numerous human infections in Cambodia during 2013. Although, human infections of H5N1 virus in Vietnam and Cambodia have declined in the recent years, further study and analysis will determine the evolutionary fitness of this genotype and its pandemic potential.

## Materials and Methods

### Virus collection, isolation, and sequencing

Pasteur Institute, Ho Chi Minh City (PI-HCMC) (Ho Chi Minh City, Vietnam) in collaboration with the Vietnamese Ministry of Health collected and analyzed samples from humans hospitalized for influenza-like illness (ILI). Informed consent to allow for swab sample collection and preliminary diagnostic testing was obtained verbally from patients. Highly pathogenic H5N1 was identified by the Institute Pasteur, Ho Chi Minh City in 4 cases from 2012 and 2 cases from 2013. Samples were shipped to the Centers for Disease Control and Prevention, Influenza Division (Atlanta, GA, USA) for virus isolation and characterization. Samples were inoculated into 10–11 day old embryonated chicken eggs. After incubation, allantoic fluid was harvested and viral RNA was isolated with Qiagen RNeasy extraction kit (Qiagen). Centers for Disease Control and Prevention Institutional Animal Care and Use Committee (IACUC) approval was not required for virus propagation in embryonated chicken eggs because all eggs were destroyed prior to hatching. All work was carried out according to guidance from the Office of Laboratory Animal Welfare (OLAW), National Institutes of Health, who is responsible for implementation of the PHS Policy Animal Welfare Act (7 U.S.C. Sections 2131–2159) and the Public Health Service Policy on Humane Care and Use of Laboratory Animals (http://grants.nih.gov/grants/olaw/faqs.htm#App_4). Influenza viral RNA was amplified using the Access Quick one-step RT-PCR kit (Promega) using H5N1 specific primers (sequence available upon request). The 400–600 bp amplicons were sequenced by using an Applied Biosystems 3730xl system using cycle sequencing dye terminator chemistry (Life Technologies). Full-length open reading frames with at least triple coverage were generated using Sequencher 5.0 (Gene Codes).

### Phylogenetic analysis

Each of the 8 viral genes was analyzed using datasets of relevant viral sequences not only from Vietnam but other Asian or European countries identified by BLAST analysis of the human sequences from Vietnam using both the GISAID (http://platform.gisaid.org) and NCBI databases (http://www.ncbi.nlm.nih.gov/nucleotide/). Sequences were aligned using the Muscle algorithm [67]. The evolutionary history of the selected viruses was inferred using the Neighbor-joining method while the evolutionary distances were computed using the Maximum Composite Likelihood method implemented in the MEGA5 software package (www.megasoftware.net) [68]. The reliability of each phylogenetic analysis was tested using bootstrap analysis with 1000 replications. Trees were either rooted to A/goose/Guangdong/1/1996 or were midpoint rooted. Gene lineages were defined using criteria described by WHO/OIE/FAO H5N1 Evolution Working Group [11,69] (for HA) or definitions previously used for analysis of H5N1 virus genotypes from Vietnam [13]. For the NA and the other internal genes, we used the nomenclature system introduced by Nguyen et al., 2012 where each lineage received a number corresponding to the gene segment (e.g. PB2: 1, PB1: 2, PA: 3, NP: 5, NA: 6, M: 7, NS: 8) and a letter to indicate the genetic lineage. We used genotype nomenclature presented in previous reports where each genotype was designated using a number after “VN” (abbreviation for Vietnam) [10,12,13]. For molecular analysis of the viral proteins, the full-length ORF sequences of NA and the internal genes starting with the ATG codon. The analysis of the HA protein used the sequence of the mature HA protein with the signal peptide removed.

### NA Inhibitors, Neuraminidase Inhibition Assay, and 50% Inhibitory Concentration Analysis

Susceptibility to the drugs zanamivir (GlaxoSmithKline, Uxbridge, UK), oseltamivir (Roche diagnostics GmgH, Mannheim, Germany), peramivir (BioCryst Pharmaceuticals, Birmingham, AL, USA), and laninamivir (compound R-125489; Biota, Victoria, Australia) was assessed by fluorescent neuraminidase inhibition (NI) assay, by using inhibitor concentration ranging from 0.03nmol/L to 1000nmol/L [70]. The 50% inhibitory concentration (IC_50_) values, the drug concentration needed to inhibit virus NA activity by 50%, were determined by using a CDC in-house program, the JASPR v1.2 curve-fitting software [71].

### Pathotyping in ferrets

#### Ethics statement

All ferret procedures were approved by Institutional Animal Care and Use Committee (IACUC) of the Centers for Disease Control and Prevention and in an Association for Assessment and Accreditation of Laboratory Animal Care International-accredited facility. Animal studies were performed in accordance with the IACUC guidelines under protocol #2234MAIFERC: "Studies on the Pathogenesis and Transmission of Recombinant Influenza Viruses in Ferrets". Male ferrets (Triple F Farms), 6 to 7 months of age and serologically negative by hemagglutination inhibition (HI) assay for currently circulating influenza viruses were used in all experiments. A minimum number of animals (3) were used to achieve reproducible results in each experiment per ethical guidelines. Ferrets were housed in cages within a Duo-Flow Bioclean mobile clean room (Lab Products, Seaford, DE) and randomly assigned to experimental groups. Temperatures were measured using a subcutaneous implantable temperature transponder (BioMedic Data Systems). Ferrets were inoculated intranasally with 10^6^ pfu of virus in a 1 ml volume (500 μl per nostril) diluted in PBS, then monitored by investigators daily for changes in body temperature, weight and clinical signs of infection. Lethargy was measured as described previously [72]. Ferrets that lost >25% of the pre-inoculation body weight or showed neurological dysfunction were euthanized based on criteria defined in the IACUC approved protocol. Following anesthesia with ketamine/atropine/xylazine solution by intramuscular inoculation into the thigh, ferrets were euthanized by direct intracardiac injection of 1 ml of Beuthanasia-D solution. Nasal washes were collected on alternate days starting on day one post-inoculation (p.i.), immediately frozen on dry ice and stored at −70°C until titration as previously described [73]. Brain and lung tissue specimens were collected for virus titration from each of three ferrets per virus at day 3 p.i. Tissues were immediately frozen on dry ice and stored at −70°C until titration as previously described [62].

### Hemagglutination inhibition assay

Antigenic characterization of HPAI H5N1 isolated from humans in Vietnam during 2012 and 2013 was performed using the hemagglutination inhibition (HI) assay with ferret antisera to a panel of representative H5N1 viruses as well as antisera generated against virus as WHO pre-pandemic H5N1 vaccine candidates. The HI assay was performed using turkey red blood cells, as previously described [74].

## Supporting Information

S1 Fig Neighbor-joining phylogenetic tree of the Polymerase basic protein 1 (PB1) gene of VN3 genotype (clade 1-like) highly pathogenic avian influenza A (H5N1) virus. Red virus strain names denote a WHO candidate vaccine virus. The 2012/2013 human cases of H5N1 from Vietnam are denoted by a green strain name. Red branching denotes human cases. Viruses previously classified with a specific Vietnam genotype are labeled parenthetically with the genotype at the end of the strain name (e.g. VN3). Bootstraps greater than 70 generated from 1,000 replicates are shown at branch nodes. The scale bar represents nucleotide substitutions per site.
(TIF)

S2 Fig Neighbor-joining phylogenetic tree of the Polymerase basic protein 1 (PB1) gene of clade VN12 genotype (2.3.2.1a-like, Hubei-like) highly pathogenic avian influenza A (H5N1) virus. Red virus strain names denote a WHO candidate vaccine virus. The 2012/2013 human cases of H5N1 from Vietnam are denoted by a green strain name. Red branching denotes human cases. Viruses previously classified with a specific Vietnam genotype are labeled parenthetically with the genotype at the end of the strain name (e.g. VN3). Bootstraps greater than 70 generated from 1,000 replicates are shown at branch nodes. The scale bar represents nucleotide substitutions per site.
(TIF)

S3 Fig Neighbor-joining phylogenetic tree of the Polymerase acid protein (PA) gene of VN3 genotype (clade 1-like) highly pathogenic avian influenza A (H5N1) virus. Red virus strain names denote a WHO candidate vaccine virus. The 2012/2013 human cases of H5N1 from Vietnam are denoted by a green strain name. Red branching denotes human cases. Viruses previously classified with a specific Vietnam genotype are labeled parenthetically with the genotype at the end of the strain name (e.g. VN3). Bootstraps greater than 70 generated from 1,000 replicates are shown at branch nodes. The scale bar represents nucleotide substitutions per site.
(TIF)

S4 Fig Neighbor-joining phylogenetic tree of the Polymerase acid protein (PA) gene of clade VN12 genotype (2.3.2.1a-like, Hubei-like) highly pathogenic avian influenza A (H5N1) virus. Red virus strain names denote a WHO candidate vaccine virus. The 2012/2013 human cases of H5N1 from Vietnam are denoted by a green strain name. Red branching denotes human cases. Viruses previously classified with a specific Vietnam genotype are labeled parenthetically with the genotype at the end of the strain name (e.g. VN3). Bootstraps greater than 70 generated from 1,000 replicates are shown at branch nodes. The scale bar represents nucleotide substitutions per site.
(TIF)

S5 Fig Neighbor-joining phylogenetic tree of the Nucleoprotein (NP) gene of VN3 genotype (clade 1-like) highly pathogenic avian influenza A (H5N1) virus. Red virus strain names denote a WHO candidate vaccine virus. The 2012/2013 human cases of H5N1 from Vietnam are denoted by a green strain name. Red branching denotes human cases. Viruses previously classified with a specific Vietnam genotype are labeled parenthetically with the genotype at the end of the strain name (e.g. VN3). Bootstraps greater than 70 generated from 1,000 replicates are shown at branch nodes. The scale bar represents nucleotide substitutions per site.
(TIF)

S6 Fig Neighbor-joining phylogenetic tree of the Nucleoprotein (NP) gene of VN12 genotype (clade 2.3.2.1a-like, Hubei-like) highly pathogenic avian influenza A (H5N1) virus. Red virus strain names denote a WHO candidate vaccine virus. The 2012/2013 human cases of H5N1 from Vietnam are denoted by a green strain name. Red branching denotes human cases. Viruses previously classified with a specific Vietnam genotype are labeled parenthetically with the genotype at the end of the strain name (e.g. VN3). Bootstraps greater than 70 generated from 1,000 replicates are shown at branch nodes. The scale bar represents nucleotide substitutions per site.
(TIF)

S7 Fig Neighbor-joining phylogenetic tree of the Matrix protein (M) gene of VN3 genotype (clade 1-like) highly pathogenic avian influenza A (H5N1) virus. Red virus strain names denote a WHO candidate vaccine virus. The 2012/2013 human cases of H5N1 from Vietnam are denoted by a green strain name. Red branching denotes human cases. Viruses previously classified with a specific Vietnam genotype are labeled parenthetically with the genotype at the end of the strain name (e.g. VN3). Bootstraps greater than 70 generated from 1,000 replicates are shown at branch nodes. The scale bar represents nucleotide substitutions per site.
(TIF)

S8 Fig Neighbor-joining phylogenetic tree of the Matrix protein (M) gene of clade VN12 genotype (2.3.2.1a-like, Hubei-like) highly pathogenic avian influenza A (H5N1) virus. Red virus strain names denote a WHO candidate vaccine virus. The 2012/2013 human cases of H5N1 from Vietnam are denoted by a green strain name. Red branching denotes human cases. Viruses previously classified with a specific Vietnam genotype are labeled parenthetically with the genotype at the end of the strain name (e.g. VN3). Bootstraps greater than 70 generated from 1,000 replicates are shown at branch nodes. The scale bar represents nucleotide substitutions per site.
(TIF)

S9 Fig Neighbor-joining phylogenetic tree of the Non-structural protein (NS) gene of VN3 genotype (clade 1-like) highly pathogenic avian influenza A (H5N1) virus. Red virus strain names denote a WHO candidate vaccine virus. The 2012/2013 human cases of H5N1 from Vietnam are denoted by a green strain name. Red branching denotes human cases. Viruses previously classified with a specific Vietnam genotype are labeled parenthetically with the genotype at the end of the strain name (e.g. VN3). Bootstraps greater than 70 generated from 1,000 replicates are shown at branch nodes. The scale bar represents nucleotide substitutions per site.
(TIF)

S10 Fig Neighbor-joining phylogenetic tree of the Non-structural protein (NS) gene of VN12 genotype (clade 2.3.2.1a-like, Hubei-like) highly pathogenic avian influenza A (H5N1) virus. Red virus strain names denote a WHO candidate vaccine virus. The 2012/2013 human cases of H5N1 from Vietnam are denoted by a green strain name. Red branching denotes human cases. Viruses previously classified with a specific Vietnam genotype are labeled parenthetically with the genotype at the end of the strain name (e.g. VN3). Bootstraps greater than 70 generated from 1,000 replicates are shown at branch nodes. The scale bar represents nucleotide substitutions per site.
(TIF)

S1 Table Amino acid differences identified in the mature HA1 hemagglutinin protein between clade 1.1 candidate vaccine viruses and select 2012 and 2013 viruses. (TIF)

S2 Table Molecular comparison of 2012 and 2013 human cases of H5N1 in Vietnam. (DOCX)
