# Supplementary figures and images for: Detection and Characterization of Clade 1 Reassortant H5N1 Viruses Isolated from Human Cases in Vietnam during 2013
Source: PLoS One. 2015 Aug 5;10(8):e0133867. doi: 10.1371/journal.pone.0133867 (PMC4526568; doi:10.1371/journal.pone.0133867)

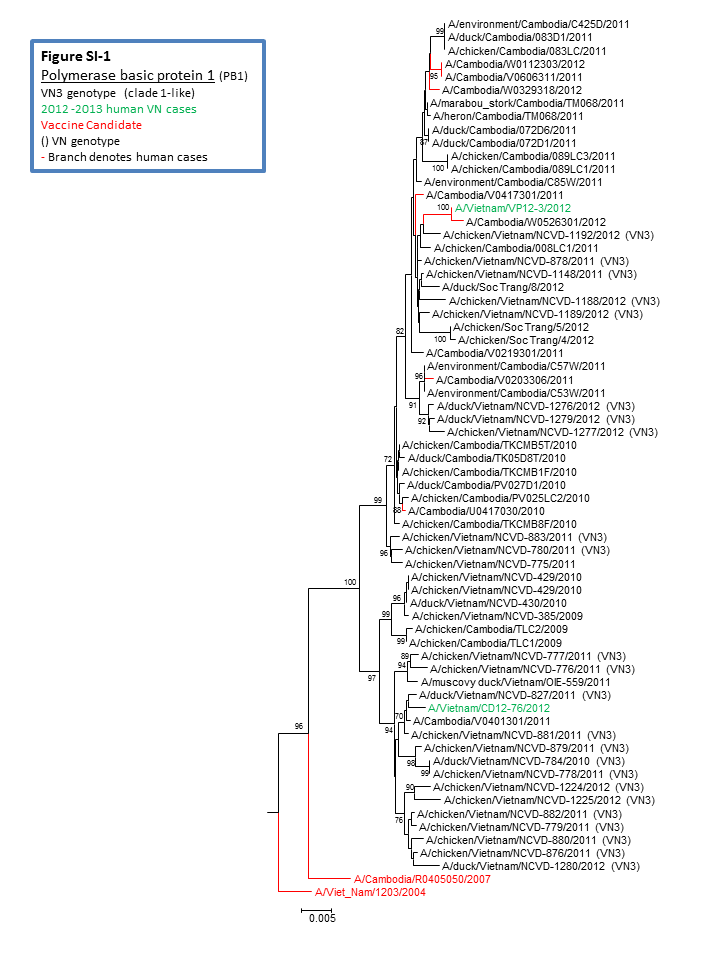

Supplement: S1 Fig — Red virus strain names denote a WHO candidate vaccine virus. The 2012/2013 human cases of H5N1 from Vietnam are denoted by a green strain name. Red branching denotes human cases. Viruses previously classified with a specific Vietnam genotype are labeled parenthetically with the genotype at the end of the strain name (e.g. VN3). Bootstraps greater than 70 generated from 1,000 replicates are shown at branch nodes. The scale bar represents nucleotide substitutions per site. (TIF) [file pone.0133867.s001.tif]

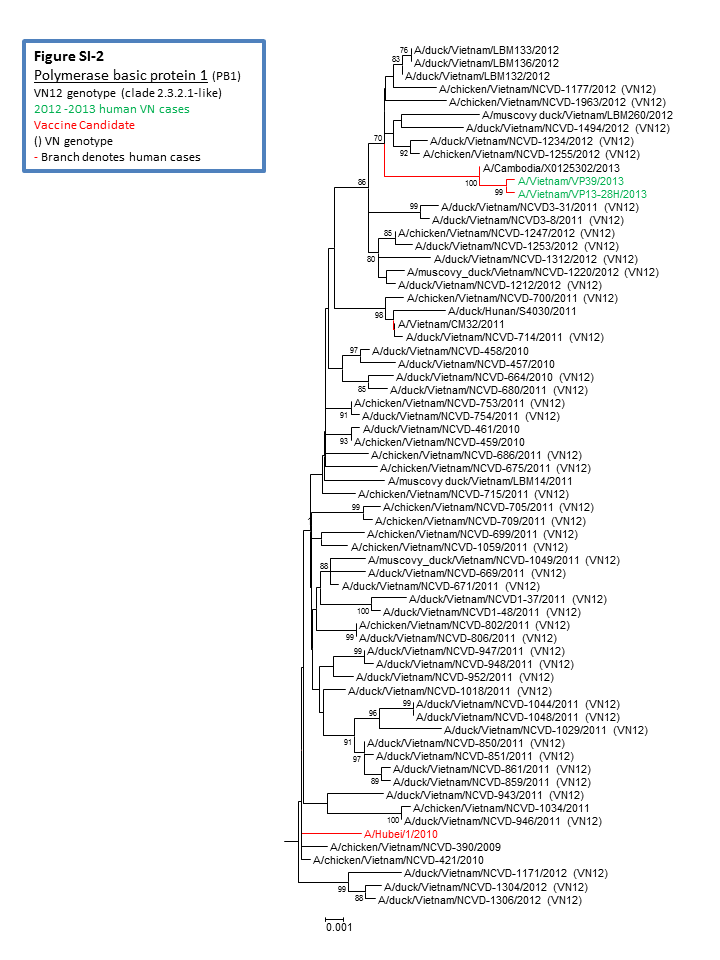

Supplement: S2 Fig — Red virus strain names denote a WHO candidate vaccine virus. The 2012/2013 human cases of H5N1 from Vietnam are denoted by a green strain name. Red branching denotes human cases. Viruses previously classified with a specific Vietnam genotype are labeled parenthetically with the genotype at the end of the strain name (e.g. VN3). Bootstraps greater than 70 generated from 1,000 replicates are shown at branch nodes. The scale bar represents nucleotide substitutions per site. (TIF) [file pone.0133867.s002.tif]

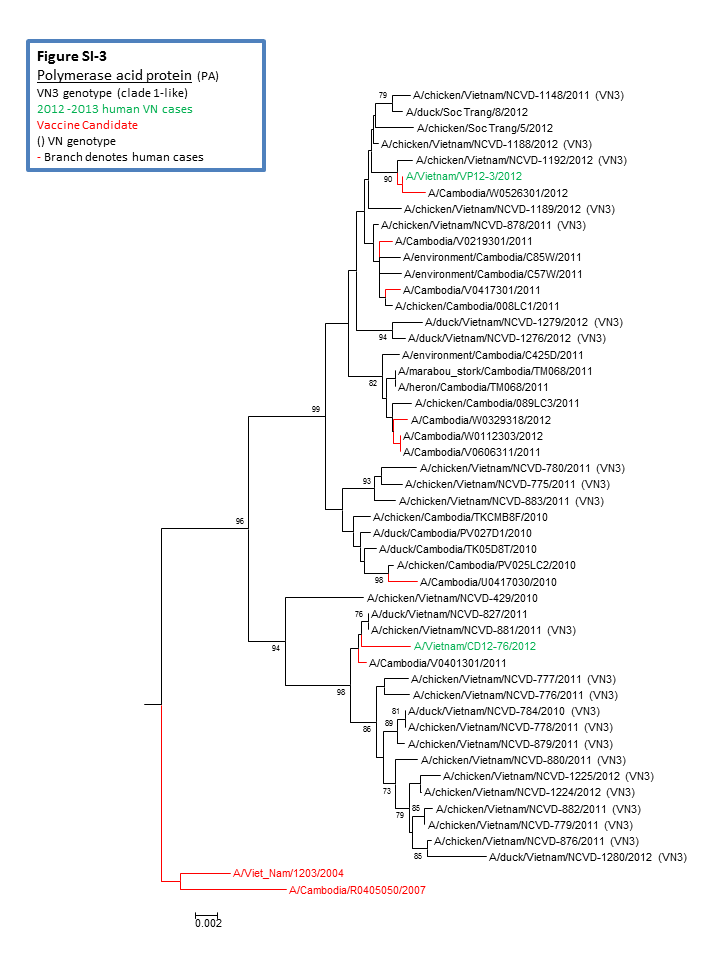

Supplement: S3 Fig — Red virus strain names denote a WHO candidate vaccine virus. The 2012/2013 human cases of H5N1 from Vietnam are denoted by a green strain name. Red branching denotes human cases. Viruses previously classified with a specific Vietnam genotype are labeled parenthetically with the genotype at the end of the strain name (e.g. VN3). Bootstraps greater than 70 generated from 1,000 replicates are shown at branch nodes. The scale bar represents nucleotide substitutions per site. (TIF) [file pone.0133867.s003.tif]

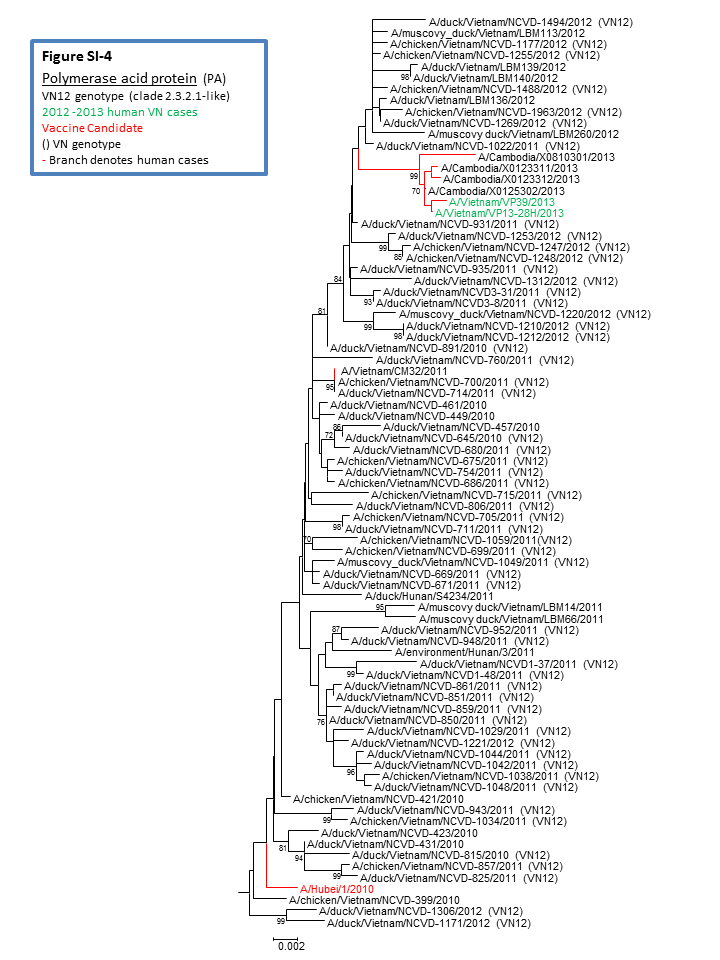

Supplement: S4 Fig — Red virus strain names denote a WHO candidate vaccine virus. The 2012/2013 human cases of H5N1 from Vietnam are denoted by a green strain name. Red branching denotes human cases. Viruses previously classified with a specific Vietnam genotype are labeled parenthetically with the genotype at the end of the strain name (e.g. VN3). Bootstraps greater than 70 generated from 1,000 replicates are shown at branch nodes. The scale bar represents nucleotide substitutions per site. (TIF) [file pone.0133867.s004.tif]

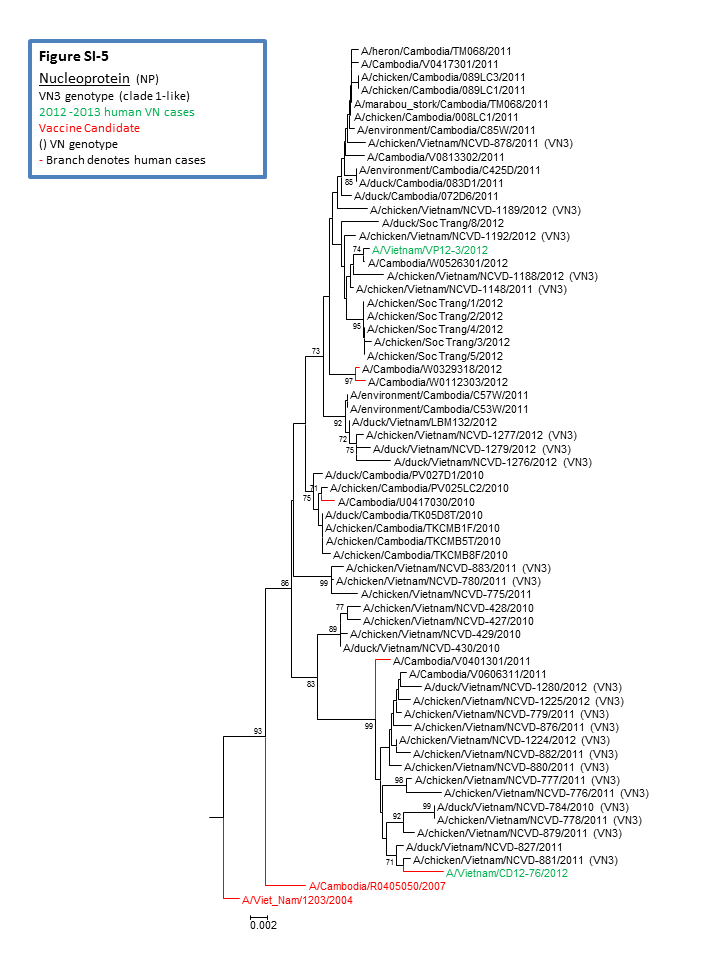

Supplement: S5 Fig — Red virus strain names denote a WHO candidate vaccine virus. The 2012/2013 human cases of H5N1 from Vietnam are denoted by a green strain name. Red branching denotes human cases. Viruses previously classified with a specific Vietnam genotype are labeled parenthetically with the genotype at the end of the strain name (e.g. VN3). Bootstraps greater than 70 generated from 1,000 replicates are shown at branch nodes. The scale bar represents nucleotide substitutions per site. (TIF) [file pone.0133867.s005.tif]

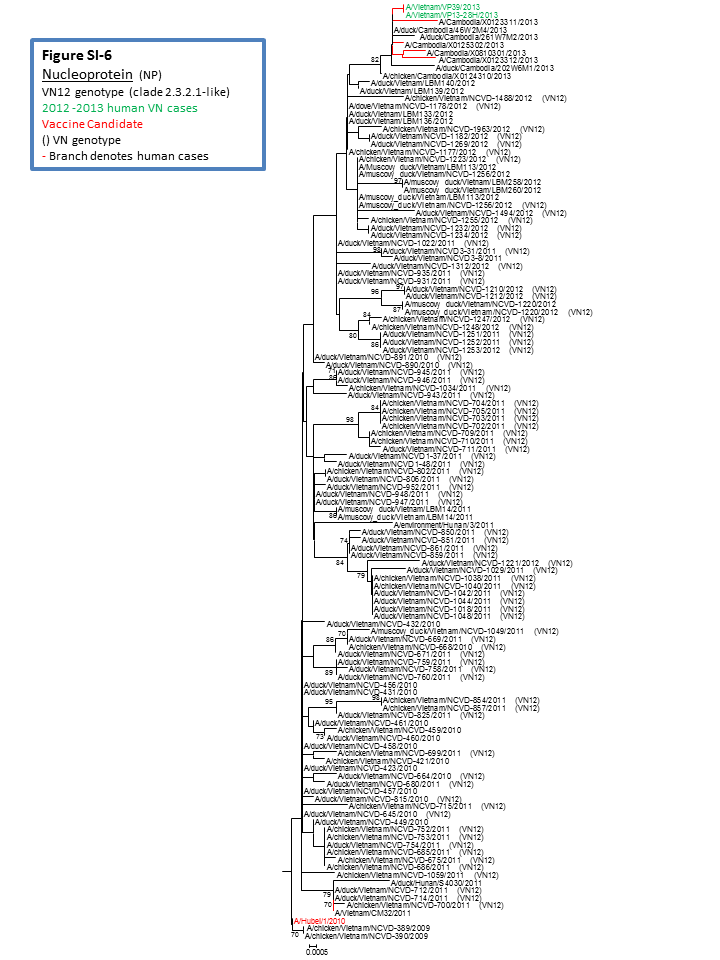

Supplement: S6 Fig — Red virus strain names denote a WHO candidate vaccine virus. The 2012/2013 human cases of H5N1 from Vietnam are denoted by a green strain name. Red branching denotes human cases. Viruses previously classified with a specific Vietnam genotype are labeled parenthetically with the genotype at the end of the strain name (e.g. VN3). Bootstraps greater than 70 generated from 1,000 replicates are shown at branch nodes. The scale bar represents nucleotide substitutions per site. (TIF) [file pone.0133867.s006.tif]

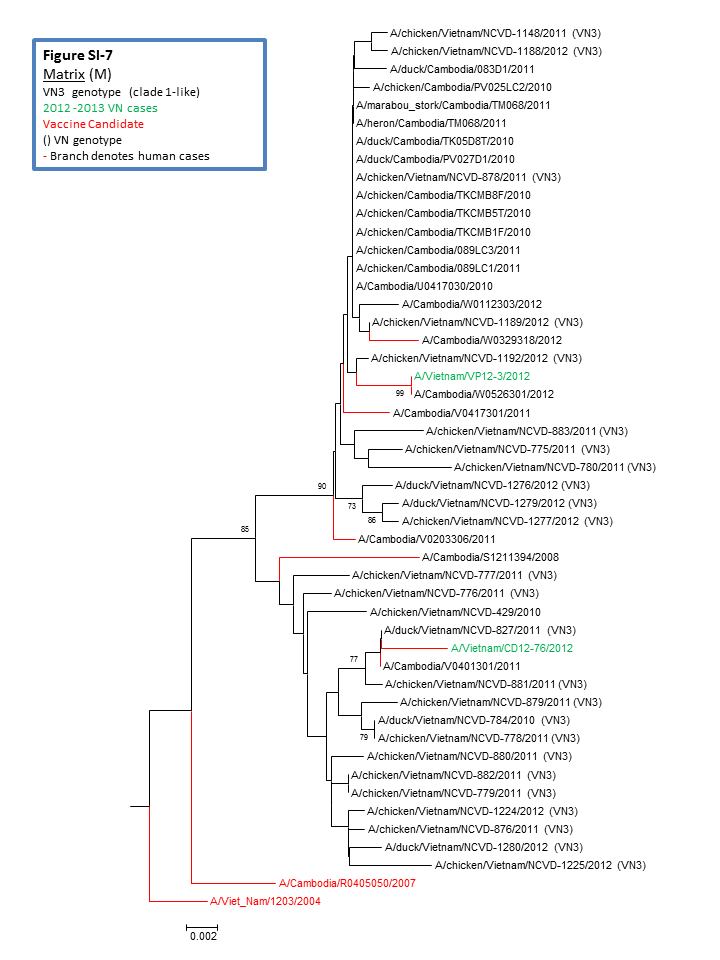

Supplement: S7 Fig — Red virus strain names denote a WHO candidate vaccine virus. The 2012/2013 human cases of H5N1 from Vietnam are denoted by a green strain name. Red branching denotes human cases. Viruses previously classified with a specific Vietnam genotype are labeled parenthetically with the genotype at the end of the strain name (e.g. VN3). Bootstraps greater than 70 generated from 1,000 replicates are shown at branch nodes. The scale bar represents nucleotide substitutions per site. (TIF) [file pone.0133867.s007.tif]

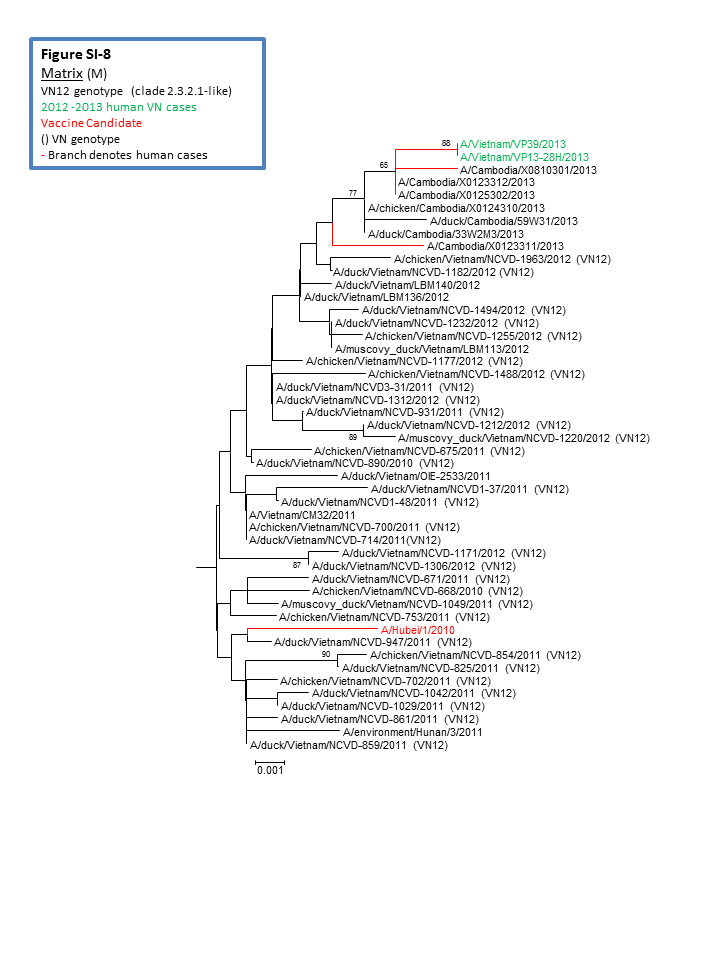

Supplement: S8 Fig — Red virus strain names denote a WHO candidate vaccine virus. The 2012/2013 human cases of H5N1 from Vietnam are denoted by a green strain name. Red branching denotes human cases. Viruses previously classified with a specific Vietnam genotype are labeled parenthetically with the genotype at the end of the strain name (e.g. VN3). Bootstraps greater than 70 generated from 1,000 replicates are shown at branch nodes. The scale bar represents nucleotide substitutions per site. (TIF) [file pone.0133867.s008.tif]

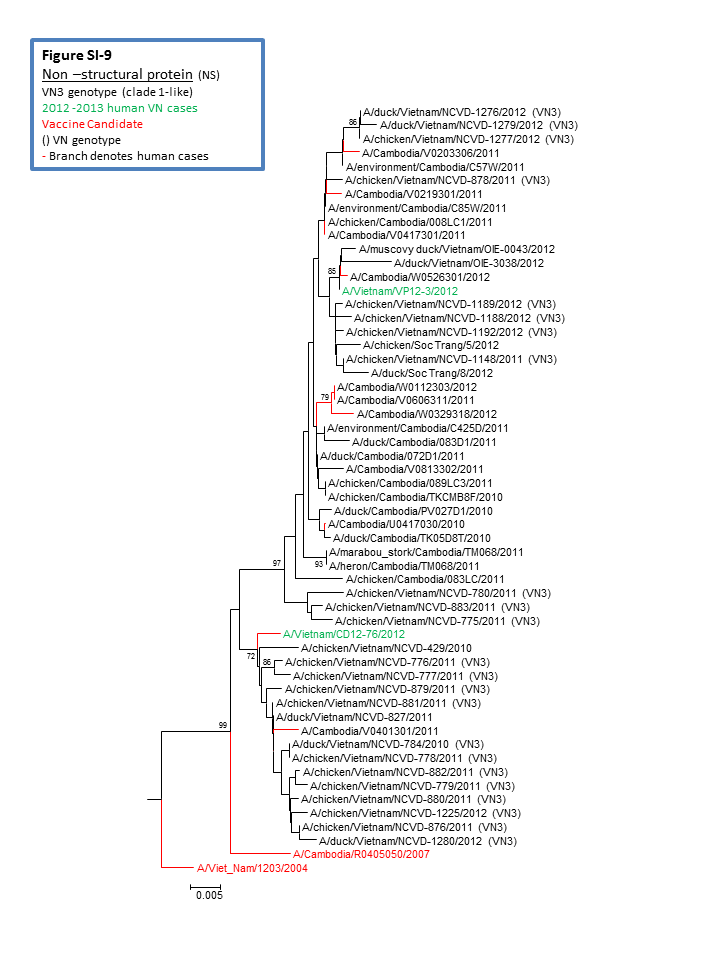

Supplement: S9 Fig — Red virus strain names denote a WHO candidate vaccine virus. The 2012/2013 human cases of H5N1 from Vietnam are denoted by a green strain name. Red branching denotes human cases. Viruses previously classified with a specific Vietnam genotype are labeled parenthetically with the genotype at the end of the strain name (e.g. VN3). Bootstraps greater than 70 generated from 1,000 replicates are shown at branch nodes. The scale bar represents nucleotide substitutions per site. (TIF) [file pone.0133867.s009.tif]

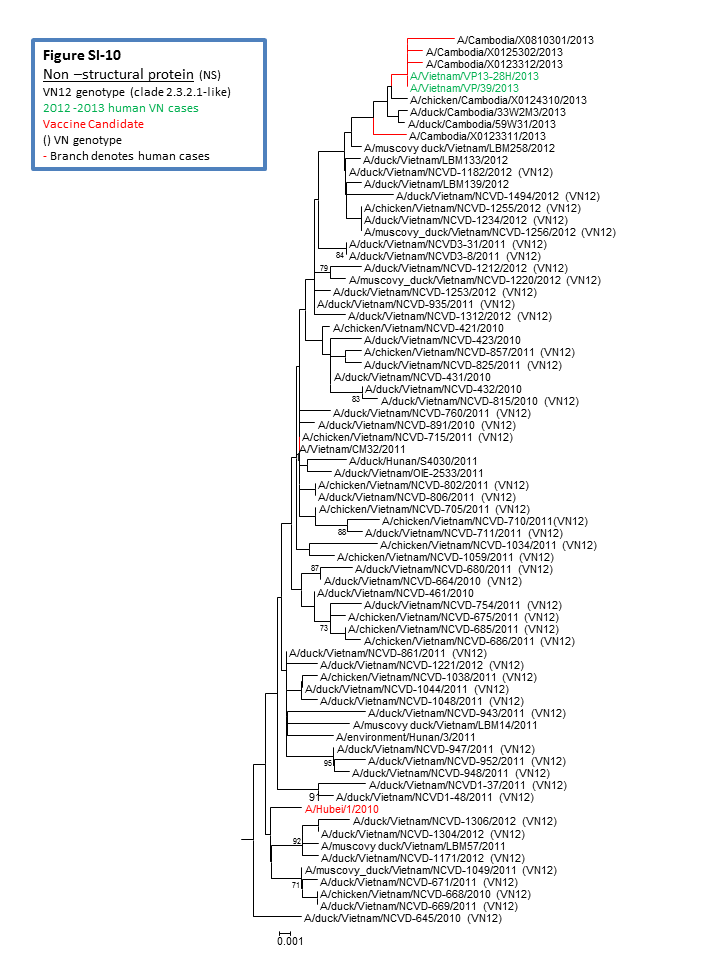

Supplement: S10 Fig — Red virus strain names denote a WHO candidate vaccine virus. The 2012/2013 human cases of H5N1 from Vietnam are denoted by a green strain name. Red branching denotes human cases. Viruses previously classified with a specific Vietnam genotype are labeled parenthetically with the genotype at the end of the strain name (e.g. VN3). Bootstraps greater than 70 generated from 1,000 replicates are shown at branch nodes. The scale bar represents nucleotide substitutions per site. (TIF) [file pone.0133867.s010.tif]

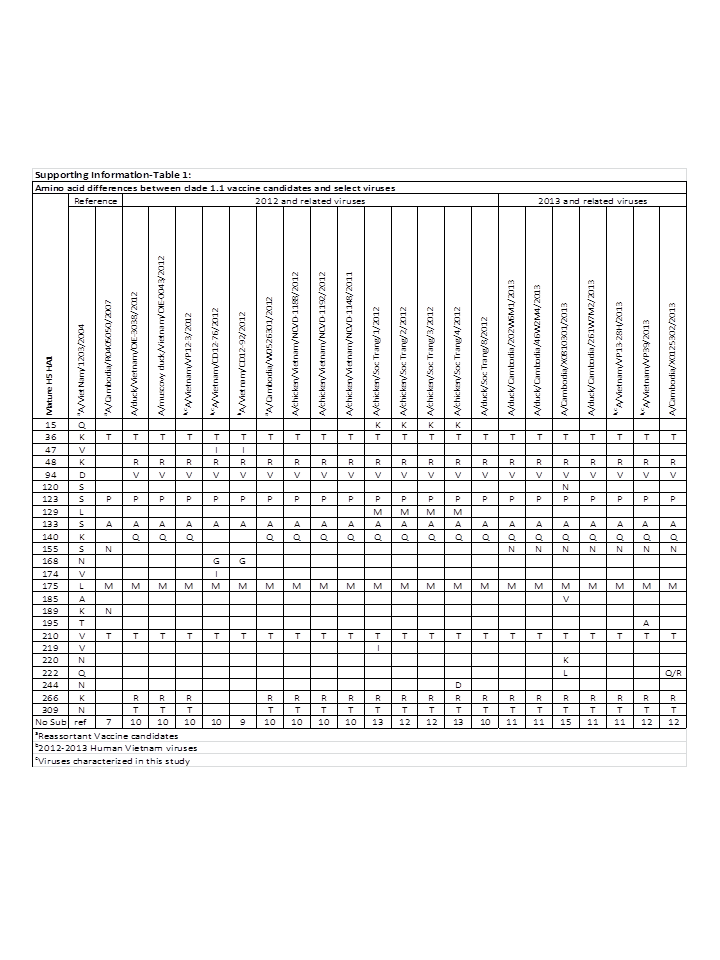

Supplement: S1 Table — (TIF) [file pone.0133867.s011.tif]
