# Supplementary material for: Detection and Characterization of Clade 1 Reassortant H5N1 Viruses Isolated from Human Cases in Vietnam during 2013
Source: PLoS One. 2015 Aug 5;10(8):e0133867. doi: 10.1371/journal.pone.0133867 (PMC4526568; doi:10.1371/journal.pone.0133867)
Supplement: S2 Table — (DOCX) [file pone.0133867.s012.docx]

Supporting Information Table 2

Molecular comparison of 2012 and 2013 human cases of H5N1 in Vietnam

| Strain | HA | | | NA | | PB2 | | PB1-F2 | | M2 | NS1 | | |
| --- | --- | --- | --- | --- | --- | --- | --- | --- | --- | --- | --- | --- | --- |
|  | MBCS^a^ | 123^b^ | 155 ^b^ | del 49-68^c^ | 129^d^ | 627^d^ | 701^d^ | Length | 66^d^ | 31^d^ | del 80-84^c^ | 92^d^ | PDZ ligand domain |
| A/Vietnam/12-3/2012 | PQRERRKKR↓G | P | S | yes | A | K | D | 90 | S | N | yes | E | ESEV |
| A/Vietnam/CD12-76/2012 | PQRERRKKR↓G | P | S | yes | V | E | D | 25 | n/a | N | yes | E | ESEV |
| A/Vietnam/VP13-28H/2013 | PQRERRKKR↓G | P | N | yes | A | E | D | 90 | N | S | yes | E | ESEV |
| A/Vietnam/VP39/2013 | PQRERRKKR↓G | P | N | yes | A | E | D | 90 | N | S | yes | E | ESEV |

^a^ MBCS: multibasic cleavage site.

^b^ Mature H5 HA numbering.

^c^ NA deletion and NS1 deletion numbering is relative to A/goose/Guangdong/1/1996.

^d^ NA and internal protein gene numbering is relative to A/Vietnam/1203/2004.
